# P29 A case of paediatric ocular Behcet’s

**DOI:** 10.1093/rap/rkab068.028

**Published:** 2021-10-19

**Authors:** Dearbhla McKenna, Diarmuid McLaughlin, Eibhlin McLoone, Cathy Campbell, Madeline Rooney, Clara McAvoy

**Affiliations:** 1 Paediatric Rheumatology, Musgrave Park Hospital, Belfast, United Kingdom; 2 Paediatric Ophthalmology, Royal Victoria Hospital, Belfast, United Kingdom

## Abstract

**Case report - Introduction:**

Bechet’s is a rare chronic inflammatory multisystem disease of unknown cause. It is characterised by recurrent oral ulcerations, genital ulcerations, ocular inflammation and skin involvement. Bechet’s disease is rarely observed during childhood and the clinical picture can be incomplete, thus making the diagnosis challenging.

Ocular involvement represents 10—20% of the initial presenting feature. Ocular involvement typically involves anterior and/or posterior uveitis and retinal vasculitis. Severe vision loss can be seen in patients.

Prognosis is improving due to enhanced therapeutic options. The use of biologics, particularly anti-TNF drugs, are a positive advance, though more evidence in paediatrics is required.

**Case report - Case description:**

This young man presented in 2014 at 12 years old to paediatric ophthalmology with a right panuveitis. He was treated with a weaning dose of oral steroids. His right eye became quiet. He reported some oral ulceration, not recurrent, and no genital ulcers. He did not fulfil Bechet’s criteria. Pathergy test and HLA B51 were negative.

In 2017 he presented with right macular retinal infiltrate. Vision was reduced to counting fingers. Mycophenolate mofetil (MMF) was started with weaning prednisolone. Despite this, activity was ongoing with inflammation in the anterior chamber and vitreous. Humira was started in August 2017. There was resolution of the inflammation in the right eye but central vision remained poor due to irreversible macular damage. Left eye was normal. Oral steroids were weaned.

In 2018, he developed acne and folliculitis type lesions. He reported sore joints and fatigue. He was felt to have probable Behcet’s. Quaternary review by the Bechet’s centre, Liverpool, agreed. A switch from MMF to azathoprine (AZA) was advised. Interferon alpha would have been suitable, but was no longer available. Skin lesions returned when switched to AZA; patient requested switch back to MMF.

Over the next 2 years steroids were weaned. MMF and 2-weekly Humira continued. In November 2020 he presented with peripheral retinitis in his left, better-seeing eye, haemorrhaging, with patchy vision. MMF was increased and oral steroids recommenced. In December 2020 visual acuity was hand movements in his right eye and 6/7.5 in his left eye with ongoing inflammation. Adalimumab was increased to weekly.

In January 21 there were 3 + of cells in his anterior chamber, extensive haemorrhages and retinal infiltrates in keeping with Behcet’s. He was admitted for pulsed IV methylprednisolone and switched from Humira to infliximab. Eyes are currently quiescent and prednisolone is weaning.

**Case report - Discussion:**

Bechet’s disease is a multiorgan disease typified by an immune-mediated occlusive vasculitis. The aetiology of Bechet’s remains unclear. HLA-B51 is a major predisposing genetic factor. There is involvement of the innate immune system, propagated by the adaptive immune system.

Ophthalmic involvement can be the presenting feature of Bechet’s. Eye disease in Bechet’s is most commonly a non-granulomatous uveitis with necrotising obliterative retinal vasculitis. This can be found in either the anterior or posterior segment or both. Recurrent inflammation in the posterior segment can lead to severe sight loss. Young males typically have the worst ocular prognosis.

The treatment of ocular Bechet’s can be challenging as our case emphasises. Treatment follows a step-wise approach. Therapy consists of topical steroid, followed by intravenous or oral steroid. Maintenance therapy of either AZA 2mg/kg/day, MMF (max3 g daily) or ciclosporin max 5mg/kg/day should be commenced. The next step will typically be a TNF inhibitor; in our patient we used adalimumab initially. Infliximab is becoming a popular first choice anti-TNF. Certainly since January 2021 our patient’s eyes have remained quiescent on Infliximab. The alternative to infliximab is interferon alpha but unfortunately this is no longer commercially available. A further treatment that could be offered is rituximab – initially to be given as a one-off cycle.

Optimum treatment of ocular Bechet’s involves good multidisciplinary team working between ophthalmology and rheumatology. By early initiation of systemic treatment and regular reviews secondary complications may be avoided/lessened.

**Case report - Key learning points:**

1. Clinical presentation of Bechet’s disease. Initially this patient presented with eye disease. He did describe oral ulceration – but not as much as 3-times per year – and he rarely had oral ulceration when examined. No other systemic symptoms of Bechet’s were present. Initial ICBD criteria was two. As time passed other clinical features presented. This case makes it important to revisit diagnosis – particularly when other symptoms become apparent.
2. Immunosuppressant Treatment in Ocular Bechets. Treatment protocols are now becoming more robust for the management of ocular Bechet’s. With more clinical trials and more evidence in adult and paediatric practice we should be able to deliver more high-quality care to these patients and thus avoid devastating complications of Bechet’s disease.
3. Use of corticosteroids. Involvement of the posterior segment of the eye with Behcet’s requires addition of steroid sparing therapy. Systemic steroids are useful to gain control of a rapidly deteriorating situation while waiting for systemic therapy to take effect and then weaned to avoid side effects.
4. Treatment Compliance. Vison loss in Bechet’s is a complication. Any chronic disease in adolescents is challenging. Taking significant medications, with side effects, that require regular blood monitoring can be cumbersome for some adolescents. This can be especially true in eye disease when for periods of time patients can be asymptomatic. We do believe, however, that it is important to counsel children and young adults and their parents of the potential risks of noncompliance/non-treatment. Young people should be aware why it is so vital to comply with medications prescribed and be encouraged to speak with their clinician if they are unable to do so.

